# First-Session Therapeutic Relationship and Outcome in High Risk Adolescents Intensive Group Psychotherapeutic Programme

**DOI:** 10.3389/fpsyg.2022.916888

**Published:** 2022-06-16

**Authors:** Kirsten Hauber, Albert Boon

**Affiliations:** ^1^LUMC Curium, Child and Adolescent Psychiatry, Leiden University Medical Centre, Leiden, Netherlands; ^2^Parnassia Psychiatric Institute, Youz Child and Adolescent Psychiatry, The Hague, Netherlands

**Keywords:** therapeutic alliance, therapeutic alliance in the initial phase, treatment outcome, psychotherapy, group therapy, adolescents’, personality disorder, MBT

## Abstract

**Background:**

An important determinant of therapy outcome is the quality of the therapeutic relationship. This study evaluated the association between the client’s assessment of first-session therapeutic relationship (FSTR) and outcome in an intensive treatment for adolescents with personality disorders.

**Method:**

Patients (N = 92) were measured weekly during intensive group treatment. The therapeutic relationship was measured with the Child version of the Session Rating Scale (C-SRS) that was completed after each group therapy session by the patient. Outcome was measured with the Child version of the Outcome Rating Scale (C-ORS). Reliable change index (RCI) was calculated for the both instruments to determine significant changes in therapeutic relationship and outcome.

**Results:**

A good FRST gave twice as much chance of a significantly better outcome. Especially for those with moderate FSTR, establishing and maintaining a good working relationship during treatment could increase the chances of a good outcome considerably. In contrast, adolescents with low FSTR had little chance of positive outcome regardless of any improvement in the therapeutic relationship.

**Conclusion:**

Adolescents assessment of FRST is indicative of the chance of a good outcome.

## Introduction

The positive association between the therapeutic relationship and outcome is demonstrated to be robust for both adults and adolescents (Flückiger et al., 2018; Karver et al., 2018; Norcross and Lambert, 2018; van Benthem et al., 2020). This therapeutic relationship remains consistent across various variables such as assessor perspectives, alliance and outcome measures, treatment method, patient characteristics, and countries (Flückiger et al., 2018). Since the effect of the technical aspects of psychotherapeutic treatment turns out to be overestimated (van Os et al., 2019) the therapeutic relationship attracts more and more attention in research and clinical practice as an important working mechanism of psychotherapy which potentially can improve the outcome, especially concerning psychotherapy in severely distressed patients (Norcross and Lambert, 2018). For adolescent mental health therapists, paying attention to therapeutic alliance in general and especially at the start of the treatment may be particularly relevant as a result of distrust of adult authorities and a desire for autonomy (De Haan et al., 2013; Hauber et al., 2020). We investigated the potential role of first-session therapeutic relationship (FSTR) ratings from the clients’ perspective to serve as an early marker of treatment outcome in a high risk adolescent sample.

The therapeutic relationship—also referred to as a working alliance—is defined as a mutual collaboration and partnership between therapist and client (Bordin, 1979). Research on predictive power of and feedback into this therapeutic relationship in ongoing psychotherapy sessions has the potential to enhance treatment outcomes (Tam and Ronan, 2017) and efficacy (Janse et al., 2017), especially for more severely disturbed patients (Norcross and Lambert, 2018) and adolescents with an increased risk of treatment failure or drop-out (De Haan et al., 2013; Hauber et al., 2020). However, relatively little research has been conducted on psychotherapy among severely disturbed adolescents with multimorbidity, a group of patients that often is excluded from scientific research (Hauber et al., 2017). Therefore, high risk adolescents’ evaluation of the therapeutic relationship combined with information on treatment outcome, in order to obtain generalisable knowledge of association between the two, are needed for clinical practice.

Studies specifically looking at the influence of the quality of FSTR on treatment outcome are rare. In a recent study on adolescent and therapists’ judgement of the therapeutic alliance, FSTR had a medium and robust association with treatment outcome. Youth with substance use disorders with a strong FSTR according to both perspectives, had an eightfold odds of favourable treatment outcome compared with adolescents with a weak FSTR according to both perspectives (van Benthem et al., 2020). The association between the client’s assessment of FSTR and outcome among high risk adolescents is unstudied.

The aim of our study was to investigate the association between the therapeutic relationship at the start of treatment and outcome in a high risk adolescent sample following intensive group psychotherapy. The therapeutic relationship was measured at the end of each group psychotherapy session with the authorised Dutch version of Child-Session Rating Scale (C-SRS) (Duncan et al., 2006; Hafkenscheid et al., 2006). Outcome was measured with the authorised Dutch version of Child-Outcome Rating Scale (C-ORS) (Miller and Duncan, 2004; Duncan et al., 2006; Hafkenscheid et al., 2010). Studies evaluating the (C-)ORS and (C-)SRS have confirmed the psychometric quality and usability of the instrument, and showed an association between the therapeutic relationship and therapeutic change or outcome (Duncan et al., 2003; Campbell and Hemsley, 2009; Boon et al., 2012; Sundet, 2012; Owen et al., 2016). Based on previous studies, it was assumed, first, that there is an association between the FSTR and treatment outcome in high risk adolescents; and second, that patients with a strong FSTR will have a higher chance of a favourable outcome compared to those with a moderate or low FSTR.

## Materials and Methods

### Setting

The studied group psychotherapy was part of a five days a week structured and integrative psychodynamic group psychotherapy programme for adolescents with personality disorders of Youz, YMHC centre in The Netherlands. This adolescent clinical psychotherapy programme commonly starts as residential treatment and converts into a day treatment halfway through. It is a mentalization based treatment (MBT) programme, manualised and adapted for adolescents (Bateman and Fonagy, 2006, 2012; Hauber, 2010) facilitated by a multidisciplinary team trained in MBT. The programme differentiates from the MBT programme for adolescents in Great Britain (Rossouw and Fonagy, 2012) because of its focus on psychodynamic group psychotherapy instead of the original more individual group psychotherapy approach. The main focus of the different therapies in this programme is not only on the adolescents’ subjective experience of oneself and others, but also on the relationships and interactions with the group members and the treatment staff. The optimal group therapy size is 6 members instead of 8. Besides the weekly group psychotherapy, other (non-verbal) group therapies as well as individual- and family psychotherapy are offered. In case medication is needed in addition to the treatment, this is prescribed by a psychiatrist of the YMHC centre.

During the 1.5 h group therapy session, the group members were stimulated to focus on oneself and others mental states that underlie overt behaviour in the group. They were invited to share their problems and focus not only on what is shared but also on how things are shared by each group member and the therapeutic alliance. Conflicts or therapeutic alliance ruptures were extensively examined and discussed. In this way, group psychotherapy is a shared attentional process which strengthens mentalising capacities and interpersonal functioning. For more details and examples of the treatment programme (Hauber et al., 2019).

### Participants

The sample consisted of 92 adolescent patients who followed the programme between 2013 and 2018. All participants were referred to the facility with clinically diagnosed personality disorders according to the DSM-III (APA, 2013), because outpatient treatment had proved insufficient. Based on the diagnostic report of the referring therapist, during the intake process, experienced clinicians of the treatment team double checked the diagnostic classifications in combination with the commitment for the treatment of the patient itself and the parents. Comorbid pervasive developmental disorder and psychosis was set as an exclusion criterion. Adolescents’ mean age at the start of treatment was 17.7 (*SD* = 1.81 range = 15–22), (females 85.9%). Average duration of treatment during this study was 215.2 days (*SD* = 100.8, range = 21–640). Most of the patients (90.4%) were clinically diagnosed with a personality disorder often with comorbid axis-I disorders (mood disorder 48.5%, anxiety disorder including PTSS 57.3%, eating disorder 8.7%, ADHD 7.6%, substance dependence 3.9%, dissociative disorder 1.9% and ASD 4.8%). Of the 94 patients diagnosed with a personality disorder, 49 (52.1%) were diagnosed as Personality disorder NAO, 16 (17%) Borderline, 16 (17%) Avoidant, 2 (2.1%) Dependent, and 1 (1.1%) Antisocial. Intelligence estimated based on level of education was average to above average. Most patients 94.4% had a native Dutch background and the Dutch language was fluently spoken by all participants.

### Instruments

The C-ORS and the C-SRS (Miller and Duncan, 2004; Duncan et al., 2006) is a measure that can be used to monitor progress during (group)psychotherapy. Both measures are four item visual analogue instruments. The versions for adolescents differ from the adult version of the ORS and SRS because it uses emoticons: a smiley (positive) and a frowny face (negative) in between 10 cm line, with instructions to place a mark on each line with low estimate to the left and high to the right. The C-ORS and C-SRS know an authorised Dutch version (Hafkenscheid et al., 2006), which has already been used in Dutch research (Boon et al., 2012; De Haan et al., 2014; Hauber et al., 2020).

The C-ORS assesses areas of life functioning known to change as a result of psychotherapy prior to the start of the treatment session. These areas are symptom distress, interpersonal well-being, social role, and overall well-being. The reliability (internal consistency) of the Dutch version of the C-ORS was satisfactory (Cronbach’s α = 0.84) (Hafkenscheid et al., 2010). The scores on these four items (the 10 cm line represents scores between 0 and 10) result in a total session score, varying between 0 and 40. This means that a high average total score indicates a low symptom distress and high well-being.

The C-SRS assesses the therapeutic relationship at the end of the psychotherapy session. This therapeutic alliance of the C-SRS consists of three interacting elements: (1) the relational bond between the patient, therapists and the group members; (2) concordance on the goals of psychotherapy; and (3) concordance on the tasks of psychotherapy. The first item assesses the feeling of being listened to; the second item assesses if the discussed topics in the session was evaluated as relevant for the patient; the third item evaluates the way the patient was approached by the therapists and group members; and the fourth and last item asks to evaluate the total session and feeling of belonging to the group. The scores on these four items (the 10 cm line represents scores between 0 and 10) result in a total session score, varying between 0 and 40. This means that a high average total score indicates a high quality of the therapeutic relationship. The reliability (internal consistency) of the Dutch version of the C-SRS was satisfactory (Cronbach’s α = 0.86) (Hafkenscheid et al., 2010).

### Procedure

At the start of the programme, all patients (N = 92) and their parents were requested permission by means of a consent form to use their data anonymously for scientific research after a verbal explanation of the treatment protocol. This written informed consent was obtained according to legislation, the institution’s policy, and Dutch law (Eurec, 2017). All subjects (N = 92) agreed to participate, and, in concordance with the institutional policy, they took part without receiving any incentives or rewards. All procedures in this study were aligned with the 1964 Helsinki declaration and its later amendments, or with comparable ethical guidelines.

The C-ORS was offered to the patients at the start of each weekly group therapy session and the C-SRS at the end of the session, after which it was collected and viewed by the therapist. According to protocol the patients were to fill in the forms during every therapy session. Although therapists sometimes forgot to hand out the C-ORS and C-SRS, the C-ORS and C-SRS were completed during most of the group therapy sessions. The first C-ORS and C-SRS were completed during the first therapy session. The C-ORS and C-SRS that were completed during the last session (planned in the case of completers and unplanned in the case of dropouts), were marked as the last C-ORS and C-SRS. It largely depended on the length of therapy how many C-ORS and C-SRS forms the patient finally completed.

### Statistical Analyses

All analyses were performed using the SPSS, version 25.0 (IBM, 2017). First, the reliable change index (RCI) was determined to calculate reliable change between the first and last C-ORS session and C-SRS session using the Jacobson and Truax formula (Jacobson and Truax, 1991) with a 95% reliability interval. Based on all questionnaires (C-ORS N = 2174; C-SRS N = 2313) for the C-ORS a reliability (Cronbach’s Alpha) = 0.842 and *SD* = 6.19 was found, the standard error was 3.48. This resulted in a reliable change criterion for the C-ORS of (1.96 × 3.48) 6.82. In case of the C-SRS the reliability was (Cronbach’s Alpha) = 0.916 and *SD* = 7.23 and the standard error 2.96. The reliable change criterion for the C-CRS was (1.96 × 2.96) 5.81.

Second, percentages of significant changes (using RCI) in the C-ORS and C-SRS between the first and last session of therapy were calculated for both the C-ORS and C-SRS.

Third, the odds ratio was calculated of the chance of a favourable outcome (C-ORS) if post-treatment the therapeutic alliance had grown (C-SRS) compared to the rest of the sample. Therefore the participants with lower scores at post-treatment on the C-SRS and the participants of which the C-SRS scores were unchanged at post-treatment, were combined.

Last, based on the FSTR (C-SRS: *M* = 26.45, *SD* = 7.23) groups were formed, namely a Low FRST group (*M* −1 *SD* ≤ 19.22), a Moderate FRST group (between *M* − 1 *SD* and *M* + 1 *SD* = 19.23–33.68) and High FRST group (*M* + 1 *SD* ≤ 33.68) (See Table 2).

## Results

### Descriptives

The 92 subjects attended group psychotherapy between March 2013 and October 2018, with an average number of group members of 5.0. The number of sessions the participants attended ranged from 9 to 44 times (*M* = 28.77, *SD* = 9.48). The number of C-ORS and C-SRS completed per participant ranged for the C-ORS from 6 to 44 (*M* = 25.35, *SD* = 8.96) and for the C-SRS from 6 to 44 (*M* = 25.12, *SD* = 8.80). Over 2,647 attended sessions, the response percentage per patient for the C-ORS ranged from 30 to 100% (*M* = 88.12%, *SD* = 13.86) and for the C-SRS from 40 ti 100% (*M* = 87.35%, *SD* = 13.18). A significant (*p* < 0.001) but moderate association was found between the C-ORS and C-SRS scores per session (*n* = 2,265, *r* = 281).

#### The Association Between the FSTR and the Outcome

First we compared the first and last session’s scores of the C-ORS and the C-SRS. Both the C-ORS (t1: *M* = 16.00, *SD* = 6.19; t2: *M* = 23.01, *SD* = 9.76; *t* = 6.54, *p* < 0.001) and C-SRS scores (t1: *M* = 26.50, *SD* = 7.23; t2: *M* = 31.49, *SD* = 10.23; *t* = 4.15, *p* < 0.001; *r* = 0.253) were significant higher at post-treatment than at pre-treatment. Table 1 shows the number and percentage of participants that deteriorated, stayed unchanged or improved on the Reliable Change Index (RCI) between first- and last session scores C-ORS and C-SRS.

**TABLE 1:** The number and percentage of participants that deteriorated, stayed unchanged or improved on the Reliable Change Index (RCI) between first- and last session scores on general functioning (C-ORS) and therapeutic alliance (C-SRS).

General functioning	Deteriorated		Unchanged		Improved		Total	
				
Therapeutic alliance	n	%	n	%	n	%	n	%
Deteriorated	2	2.1	4	4.3	2	2.2	8	8.6
Unchanged	3	3.3	23	25.0	16	17.4	42	45.7
Improved	1	1.1	18	19.6	23	25.0	42	45.7
Total	6	6.5	45	48.9	41	44.6	92	100.0

*RCI, Reliable Change Index; C-ORS, Child Outcome Rating Scale; C-SRS, Child-Session Rating Scale.*

**TABLE 2:** Comparison of number and percentage of not improved and improved participants between first- and last session scores on general functioning (C-ORS) and quality of the FSTR (C-SRS).

General functioning	Not improved		Improved		Total		
			
	n	%	n	%	n	*OR*	*p*
Low FSTR not improved	2	100.0	0	0.0	2		
Low FSTR improved	6	85.7	1	14.3	7	1.167	0.571
Moderate FSTR not improved	25	71.4	10	28.6	35		
Moderate FSTR improved	13	37.1	22	62.9	35	4.231	0.004

*FSTR, First-session therapeutic relationship.*

Second, the association between the therapeutic relationship and the outcome was investigated. In general, if the therapeutic alliance stayed unchanged (C-SRS), just over a quarter (28.6%) of the participants had a significantly better outcome (C-ORS). However, if the therapeutic relationship did improve, almost 63% had a significantly better outcome at the end of treatment. In the case that at post-treatment the therapeutic alliance had grown, the chance of a favourable outcome was more than twice as large (*OR* = 2.152 95% CI 0.931–4.976).

#### The Patients’ Assessment of the FSTR and the Chance of a Favourable Outcome

Of the total of 92 participants, 9 (9.8%) reported a low (*M* − 1 *SD*) therapeutic alliance in the first session of the treatment, 70 (76.1%) a moderate (between *M* − 1 *SD* and *M* + 1 *SD*) therapeutic alliance and 13 (14.1%) a high (*M* + 1 *SD*) therapeutic alliance. Of the Low FRST group (N = 9), the therapeutic relationship improved in 7 (77.8%) cases, but only 1 (11.1%) had a favourable outcome (*p* = 0.571). In contrast, the therapeutic alliance did not improve for anyone in the High FRST group (N = 13) and 8 (61.5%) had a favourable outcome. When in the Moderate FRST group (N = 70) the therapeutic alliance did not improve, only 37.1% (n = 13) had a favourable outcome; but when the therapeutic alliance did improve, 62.9% (n = 22) recovered significantly (*p* = 0.004).

In case a post-treatment therapeutic alliance had improved in this Moderate FRST group, the chance of a significantly better outcome was more than four times as high (*OR* = 4.231 95% CI 1.550–11.546). In contrast, the chance of a favourable outcome in the Low FRST group when the therapeutic alliance had improved was 1.167 (95% CI 0.862–1.579) (See Table 2).

## Discussion

The aim of our study was to gain deeper insights on the association between the first-session quality of the therapeutic relationship and treatment outcome among high risk adolescents receiving intensive MBT. We measured the therapeutic relationship with the C-SRS and the outcome with the C-ORS during clinical adolescent’s group therapy. As expected, in general a good FSTR gave twice as much chance of a significantly better outcome (*OR* = 2.2). In case the therapeutic alliance did not improve, just 36.0% of the respondents (*N* = 18) had a significantly better outcome while if the therapeutic relationship did improve, 54.8% (*N* = 23) had a significantly better outcome. Especially for those with moderate FSTR, establishing and maintaining a good working relationship during treatment could increase the chances of a good outcome considerably (*OR* = 4.2). In contrast, adolescents with low FSTR had little chance of positive outcome regardless of any improvement in the therapeutic relationship (*OR* = 1.2). This could mean that clinical adolescents’ assessment of FRST is indicative of the chance of a good outcome. Our study showed that the rather short instrument (C-SRS), which can be easily applied in clinical practice to be completed by adolescent patients themselves, is a valuable instrument for measuring the quality of the therapeutic relationship.

The results of this study provide evidence concerning the significance of the FSTR and of the client-therapist match in high risk adolescents. In the intake process it seems crucial to establish a good quality therapeutic relationship to increase the chance of an average or high treatment outcome. Maybe for high risk adolescents with personality disorders and insecure attachment (Hauber et al., 2018) an intense focus on the therapeutic relationship from the start of treatment is extra helpful in establishing and maintaining alliance (Groth and Hilsenroth, 2019; Hauber et al., 2020). Therapeutic ruptures can quickly be repaired and drop out of treatment prevented (Hauber et al., 2020).

In the light of psychotherapies’ equivalent paradox—‘treatments have equivalently positive outcomes despite non-equivalent theories and techniques’ (Stiles et al., 2008)—FSTR could help enhance treatment outcomes and the (cost-)effectiveness of psychotherapy for adolescents with personality disorders. As adolescents with low FSTR had little chance of positive outcome regardless of any improvement in the therapeutic relationship, it is worth considering stopping the treatment in consultation with the patient and family. A frank discussion with the patient and their parents about the low probability of a positive outcome provides an opportunity to adjust or stop the treatment, and to look for a more suitable treatment.

## Conclusion

In this study, the association between the quality of the FSTR from the patients’ point of view and treatment outcome was examined in a seldom studied adolescent group with personality pathology. Personality disorders often manifest themselves in mid to late adolescence for the first time (Kessler et al., 2005). Despite this knowledge, research and clinical attention is focussed mainly on adults and then mainly on borderline personality disorder. Against this background, clinical practice is in need of more information on this difficult patient group. Research investigating moderators of outcome among psychotherapy treatments for adolescent personality disorders is needed. Understanding for whom, and under what conditions and in which dosage, treatments exert their greatest effects is essential and enhances development of personalised psychiatry. Furthermore, the role of parents and peers could be an important factor of influence on the outcome of intensive treatment and needs further study.

Limitations of this study must be mentioned. The first limitation is that it is not clear if these results found in a sample of high risk adolescents can be generalised to (group) psychotherapy with other patients with personality pathology and patients with other pathology. The second limitation is that Axis-I disorders were left out due to the practical consideration of not overloading patients with assessment instruments. Nevertheless, the C-SRS can help psychotherapists estimate the change of a positive treatment outcome.

## Data Availability Statement

The raw data supporting the conclusions of this article will be made available by the authors, without undue reservation.

## Ethics Statement

Ethical review and approval was not required for the study on human participants in accordance with the local legislation and institutional requirements. The patients/participants provided their written informed consent to participate in this study.

## Author Contributions

KH performed the data collection and wrote the manuscript. AB contributed to the design of the research project, performed the statistical analyses in the study, and revised the manuscript. Both authors read and approved the final manuscript.

## Conflict of Interest

The authors declare that the research was conducted in the absence of any commercial or financial relationships that could be construed as a potential conflict of interest.

## Publisher’s Note

All claims expressed in this article are solely those of the authors and do not necessarily represent those of their affiliated organizations, or those of the publisher, the editors and the reviewers. Any product that may be evaluated in this article, or claim that may be made by its manufacturer, is not guaranteed or endorsed by the publisher.
